# Activation of tonsil dendritic cells with immuno-adjuvants

**DOI:** 10.1186/1471-2172-9-10

**Published:** 2008-03-18

**Authors:** Marta E Polak, Nicola J Borthwick, Francis G Gabriel, Martine J Jager, Ian A Cree

**Affiliations:** 1 Translational Oncology Research Centre, Queen Alexandra Hospital, Portsmouth, UK; 2 Department of Pathology, Institute of Ophthalmology, London, UK; 3 Department of Ophthalmology, Leiden University Medical Center, Leiden, The Netherlands

## Abstract

**Background:**

Dendritic cells (DC) play the key role in directing antigen-specific immune responses and manipulating their function may be a useful tool for immunotherapy. The balance between immune stimulation and tolerance is particularly important at mucosal interfaces, where discrimination between dangerous pathogens and innocuous antigens takes place. In humans, although much is known about the responses of monocyte derived DC, relatively little is known about effect of immuno-stimulatory adjuvants on DC found in tonsil.

**Results:**

To examine this, tonsil DC were isolated and cultured with potent DC activators; IFNγ, anti-CD40 antibody, LPS and Poly I:C either singly or in combination. To measure maturation and activation, DC were examined for changes in the expression of HLA-DR, HLA- class I, CD83, CD40, CD80 and CD86 and the release of IL12p70.

The DC isolated from tonsil were a mixed population containing both myeloid and plasmacytoid DC, but all showed similar responses. Tonsil DC released IL12p70 upon stimulation with IFNγ , anti-CD40 antibody, and LPS, but unlike monocyte-derived DC, they did not increase the expression of cell surface activation molecules above those induced by culture alone. Poly I:C, a potent stimulator of laboratory generated DC inhibited the activation of tonsil DC by other adjuvants.

**Conclusion:**

As the response of this mixed population of DC does not mirror that of DC generated *in vitro*, this may have implications for other tissue residing DC and might be an important consideration for immunotherapy.

## Background

Dendritic cells orchestrate the primary antigen specific immune response and manipulating their function could potentially benefit the treatment of many disorders including autoimmune diseases and cancers. Their primary function is to present antigen to naïve T lymphocytes and in so doing either induce or suppress the immune response. Induction occurs if antigen is recognised as potentially dangerous. This requires a second signal, such as pro-inflammatory cytokines, CD40-CD40L signalling or prostaglandins [1-3]. The DC respond to these signals by upregulating the cell-surface molecules required for efficient antigen presentation and naïve T lymphocyte activation (HLA class I, HLA-DR, CD80, CD86, CD40) and by secreting immunostimulatory cytokines, such as IL-12 [4,5]. Antigen presentation in the absence of second signals causes anergy or unresponsivenes [6-8]. Suppression of the immune response may also be achieved by the action of different subsets of DC including plasmacytoid DC or by the interaction of regulatory T cells with DC [9-12]. The outcome of antigen presentation therefore depends upon the subsets of cells and accessory signals that are present in the tissue microenvironment.

Much of our knowledge of human DC has been gained through the study of monocyte or bone marrow derived DC generated and manipulated *in vitro *(MoDC) [13]. Monocytes cultured in the presence of IL-4 and GMCSF differentiate into immature myeloid DC, characterised by high expression of HLA-DR and CD11C. Subsequently, a variety of adjuvants can be used to induce DC maturation and activation. Various adjuvants have been tested on cultures of MoDC to varying effect, but the most potent described include bacterial lipopolysaccharide (LPS), recombinant nucleotides that mimic viral infection (Poly I:C), CD40/CD40L interactions and the cytokine IFNγ [14-17].

To the contrary, data presenting activation of human tissue-derived DC, i.e. gut or respiratory tract, are very scarce and little is know how they respond to direct activation with immuno-adjuvants. Nevertheless, tissue DC have long been recognized as the key regulators of immune responses [18-22] and understanding of their biology may significantly improve management of immune related disorders as well as cancer. To investigate the response of tissue-derived DC, we created an alternative to MoDC model, using palatine tonsils, which have long been a source of human lymphoid tissue.

Tonsils protect the gateway of both respiratory and alimentary tract and their main function is to discriminate between potentially infectious pathogens and innocuous airborne and food antigens. They are particularly interesting because they are constitutively immunosuppressed as part of the mucosa-associated lymphoid tissue (MALT), but are still able to evoke a potent immune response to pathogens [22,23]. A number of DC subsets have been identified in tonsil. Like all DC they express HLA-DR in the absence of the lineage associated markers CD3, CD19, CD14 & CD16 but are distinguishable by the intensity of HLA-DR and the expression of CD11C & CD123 [24,25]. The HLA-DR+, CD11C+ subset generated from monocytes can also be identified in tonsil but it is not known if both populations function similarly.

To examine whether tonsil DC respond to adjuvant activation in a similar manner to laboratory generated DC we have isolated DC from tonsil and cultured them with a number of immune adjuvants commonly used to activate monocyte derived DC, including some which could be used therapeutically in cancer. Our results show that mixed DC populations from tonsil can be activated with adjuvants but that their responses do not directly correlate with those of monocyte-derived DC. In particular, Poly I:C appears to suppress activation and IL-12 release in response to potent adjuvant stimuli.

## Results

### Isolation of Tonsil DC

**Figure 1:**
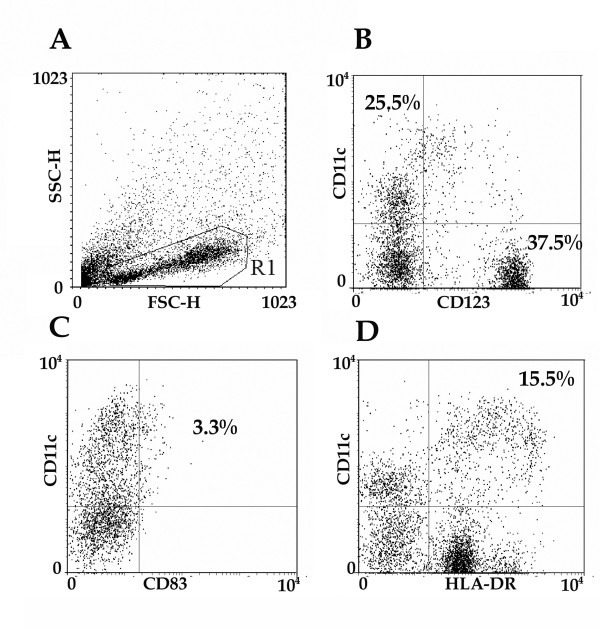
**The expression of DC markers on the tonsil DC fraction**. The DC fraction from tonsil was investigated for expression of the DC markers: CD11c (myeloid DC), CD123 (plasmacytoid DC) and CD83 (mature DC). A representative example is shown. The FSC, SSC profile (A) shows the size and granularity of the events and was used to set a region (R1) around the viable cells. This excluded dead cells, debris and any contaminating large stromal cells from the analysis. The percentages shown are the percentage of cells within R1 that fall into the quadrants.

The techniques developed for the isolation of tonsil DC allowed us to recover reasonable numbers of viable cells relatively quickly. A total of 15 tonsils were investigated. The original cell suspension contained on average 1.68 ± 0.40 × 10^9 ^cells in total. The purification process removed T and B lymphocytes, tonsil stromal cells and red blood cells and resulted in a fraction containing 8 × 10^4 ^to 1 × 10^7 ^cells, mean 2.68 ± 0.69 × 10^6^. The final cell population was highly enriched in DC. CD19, CD3 and CD14 positive cells were rare (< 0.5%). Up to 96.5% of the purified cells were HLA-DR+ (mean: 65.1% ± 6.64), and expressed CD11c (30.63% ± 4.12), CD123 (37.1% ± 5.46), CD83 (5.15% ± 0.44). Cells positive for both HLA-DR and CD11c (myeloid DC) accounted for 16.3 ± 3.55% of the purified APC fraction (Table 1). A typical example of the enriched DC fraction is shown in Figure 1. The FSC and SSC profiles show the size and granularity of the events and were used to position the live cell region (R1, Fig. 1a). Dead cells and cell debris are shown above and to the left of R1. This scatter profile shows the wide variation in size of the cells in the purified fraction. There are two main mutually exclusive populations defined by expression of either CD11C or CD123 and HLA-DR (Fig. 1b, d) neither of which express high levels of CD83 (Fig. 1c). The HLA-DR negative cells in the fraction are probably contaminating macrophages.

**Table 1:** Phenotype of APC freshly isolated from tonsils.

Dendritic cell marker	Positive cell fraction [%]
CD11c	30.63 ± 4.12 (11.96–63.51)
CD123	37.10 ± 5.46 (15.94–52.94)
CD83	5.15 ± 0.44 (4.04–5.72)
HLADR	65.10 ± 6.64 (32.15–96.46)
HLADR^high ^and CD11c+	16.3 ± 3.55 (10.94–28.37)

The numbers in the table show the mean percentage value of positive fraction from all 15 tonsils analysed, with the SEM value. The values in brackets show the range of values for each subset analyzed.

**Figure 2:**
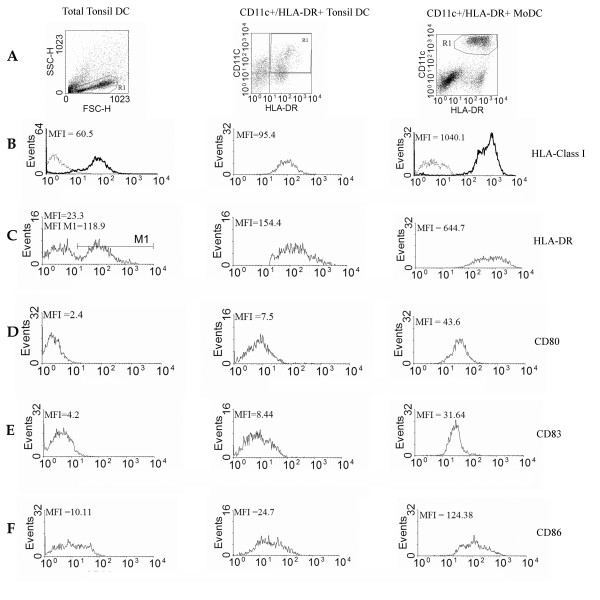
**The expression of DC activation markers on DC**. Whole tonsil DC (I) and the CD11c+/HLA DR^hi ^tonsil DC (II) and monocyte-derived DC (III) were compared for the expression of HLA-Class I (B), HLA-DR (C), CD80 (D), CD83 (E) and CD86 (F). R1 represents the region used to gate the cells of interest (A); MFI (mean fluorescence intensity); MFI M1 (mean fluorescence intensity in marker 1).

We next examined the phenotype of the tonsil DC fraction in terms of the DC activation markers: CD40, CD80, CD83, CD86 and HLA DR. As the tonsil fraction was heterogeneous and included immature myeloid DC (CD11c+/HLA-DR^med^), plasmacytoid DC (CD123+) and activated DC (CD11c+/HLA DR^hi^), described by Hart and colleagues (21), we compared the expression of DC activation markers on the whole fraction and on CD11c+/HLA DR^hi ^cells (Fig. 2). Compared to the whole fraction, the CD11c^+^/HLA-DR^hi ^DC express slightly more of each of the activation markers.

### Culture of tonsil DC with adjuvant

**Figure 3:**
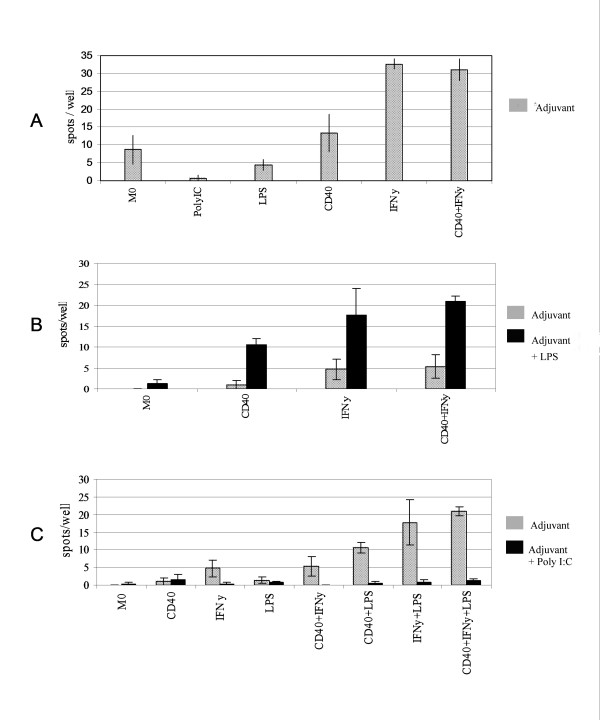
**The production of IL-12 from tonsil DC after stimulation with immune-adjuvants**. Tonsil APC were stimulated with CD40L, IFNγ, LPS and Poly IC either singly (a) or in combinations with LPS (b) or Poly I:C (c). Production of IL12 by unstimulated cells in culture (M0) was measured for comparison. The grey bars show adjuvants and adjuvant combinations alone and the black show these same adjuvants or combinations together with LPS (b) or Poly I-C (c) The bars represent mean values of three wells from one representative experiment of five, ± SEM.

We next cultured the tonsil DC fraction with a range of commonly used adjuvants previously shown to activate monocyte derived DC. These included IFNγ, a humanized monoclonal antibody against CD40 that mimics T cell interactions via CD40 ligand, GMCSF, bacterial LPS, and Poly I:C. To assess their effect we measured IL-12, a pro-inflammatory cytokine secreted by mature DC. IL-12p70, the bioactive subunit of IL-12, was measured after 36–48 hours using the ELISpot technique in tonsil DC from 5 individuals. Unstimulated cells cultured in medium alone produced low amounts of IL-12, while both anti-CD40 antibody and IFNγ proved to be strong activators of tonsil DC (Fig. 3a). The combination of anti-CD40 antibody and IFNγ increased IL-12 to levels seen using INFγ alone indicating this cytokine to be particularly potent. The addition of LPS alone did not affect IL-12 production but the combinations of LPS and anti-CD40 antibody or LPS and IFNγ induced unexpectedly high numbers of DC to produce IL-12 (Fig. 3b). The highest levels of IL-12 release were achieved when all three adjuvants were combined (Figure 3b). Sequences of nucleotides rich in cytosine and guanine have been shown to be very potent activators of monocyte-derived DC (16). However, when Poly I:C was added to tonsil DC it greatly antagonised the release of IL-12 induced by any other immune-adjuvant, and even depressed the baseline production of IL-12 in cell culture (Fig. 3c). This was not due to the death of the DC as both microscopic examination and propidium iodide/annexin V studies failed to show any evidence of apoptosis or excessive loss of viability using poly I:C (data not shown).

### Changes in antigen expression

**Figure 4:**
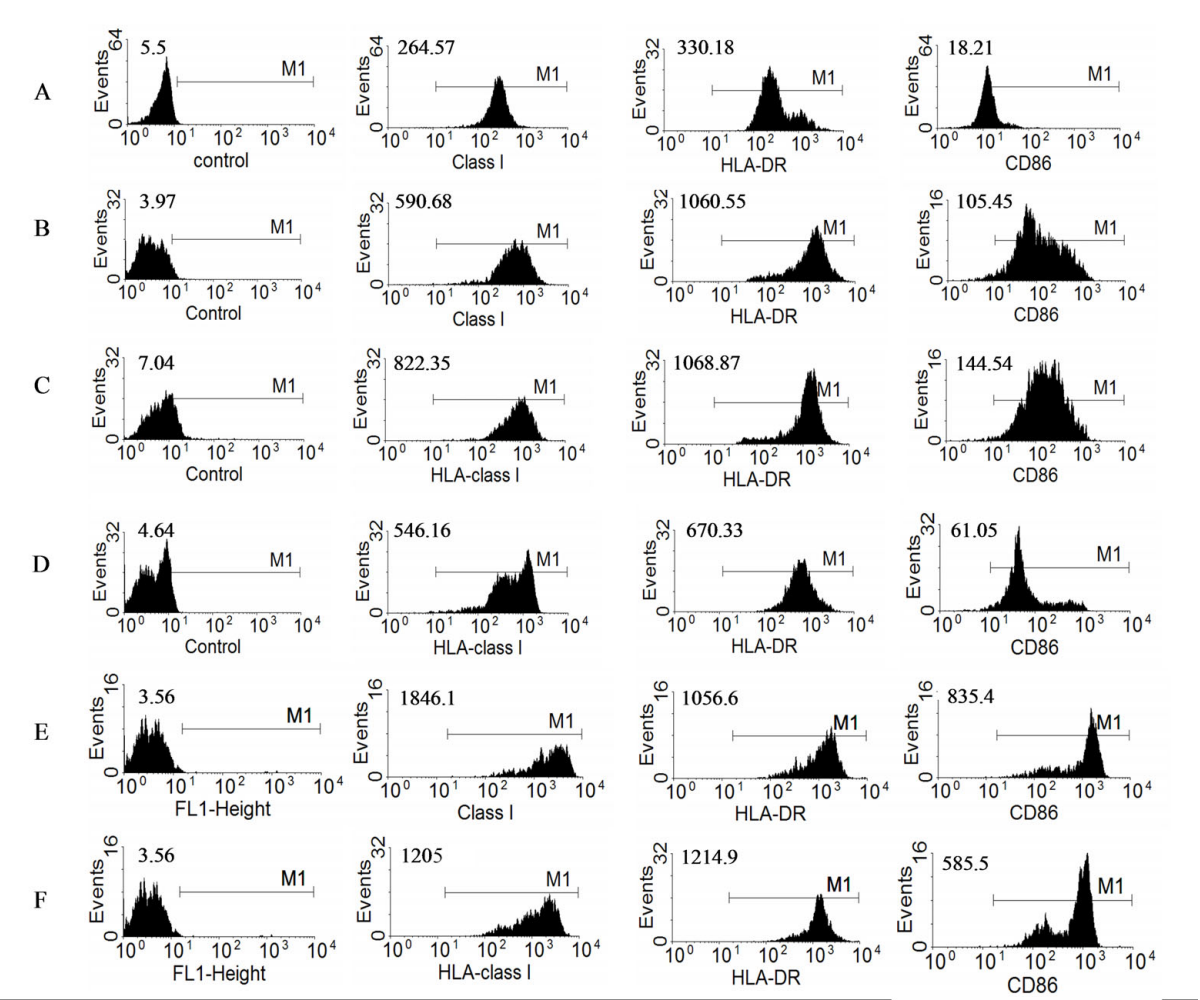
**Changes in DC surface antigen expression after stimulation with immune-adjuvants**. CD11c^+^/HLA DR^hi ^tonsil DC (a-d) and blood monocyte-derived DC (e-f) were investigated by flow cytometry before stimulation (a), after overnight culture in medium alone (b) and after culture with anti-CD40, IFNγ and LPS (c & e) or anti-CD40, IFNγ, LPS and Poly I:C (d & f). The isotype control was used to set the marker M1. The MFI of the cells in MI is shown on the left. The data shown comes from one representative experiment of ten performed.

Activated DC express increased amounts of antigen presenting molecules and co-stimulatory molecules on their surface that facilitate efficient lymphocyte activation. We therefore examined the changes in surface antigen expression on tonsil and monocyte derived DC following culture with adjuvants. In order to compare the CD11c+/HLA DR^hi ^activated DC these were gated during the data analyses. (Figure 4 a–d). Compared to monocyte derived DC, tonsil CD11c+/HLA DR^hi ^DC showed a less marked response to the adjuvants investigated. The culture of tonsil DC in media alone induced an up-regulation of CD86, HLA DR, HLA class I (Fig. 4b). The response to the most potent combination of adjuvants CD40, IFNγ & LPS is shown in Fig. 4c. From this it is clear that although there is some up-regulation of class I and CD86 it is not very dramatic and does not reach the same levels as those seen on monocyte derived DC (Fig. 4e). The combination of anti-CD40 antibody and IFNγ (data not shown) had very little effect over and above culture in medium alone. As observed for IL-12 production, Poly I:C suppressed tonsil DC activation in culture, in particular decreasing the expression of CD86 (Fig. 4d). This effect was observed to an even greater extent when the whole population of tonsil APC was examined (data not shown). Poly I:C also caused some cell shrinkage and we observed depletion of CD123+, HLA class ^hi ^cells (data not shown). These results were consistent for all 10 tonsils analysed.

As a control, we investigated the responses of monocyte derived DC obtained from peripheral blood. After the standard 5 days in culture with GMCSF and IL4, the monocytes differentiated into DC, expressing HLA class I ^hi^, HLA DR^hi ^& CD11C. The CD11c+/HLA DR ^hi ^cells comprised up to 80% of the cultures. These DC were then either cultured with the same immune-adjuvants as tonsil DC, left in culture with GMCSF and IL4, or transferred to culture medium for two consecutive days. We did not observe any difference between an extra 48 hours in GMCSF and IL4 over culture in media alone: the expression of CD86 increased under both conditions (data not shown). Culture of the cells in the triple adjuvant combination; anti-CD40, IFNγ & poly I:C up-regulated HLA-class I, HLA-DR and CD86 over and above the levels seen in tonsil DC (Fig. 4c &4e). Interestingly, unlike tonsil, the addition of poly I:C did not cause a dramatic decrease in any of these markers (Fig. 4f)

**Figure 5:**
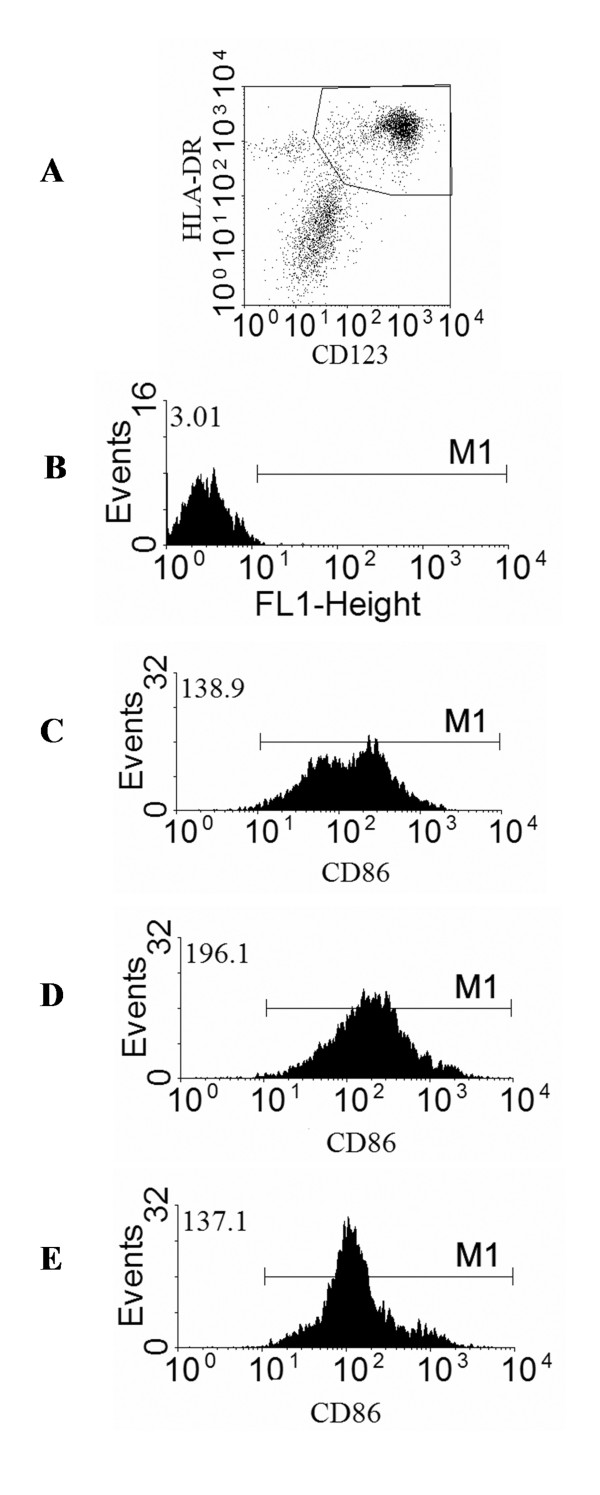
**Changes in expression of CD86 on tonsil plasmacytoid DC**. CD123^+^/HLADR^hi ^plasmacytoid DC were gated during analysis (a). Marker M1 was set using an isotype control on unactivated cells (b). CD86 was examined after activation with anti-CD40 and IFNγ (c) anti-CD40, IFNγ and LPS (d) anti-CD40, IFNγ, LPS and Poly I:C (e). The numbers in right upper corners show MFI values for region M1. The data shown comes from one representative experiment of three performed.

As plasmacytoid DC make up a proportion of the tonsil DC population we also examined the changes in expression of activation molecules by CD123+/HLA DR^hi ^cells after stimulation. Figure 5 illustrates the changes in expression of CD86 after culture with anti-CD40 IFNγ & LPS and the effect of poly I:C. Once again the adjuvant combination increased CD86 expression and poly I:C antagonised this. We did not observe any correlation between the initial proportion of each DC sub-population and the ultimate effect of the adjuvants.

## Discussion

Most knowledge of the biology of DC has been acquired from studies of monocyte or blood precursor derived DC, generated and matured *in vitro*. However, the biology of DC residing in tissues may be more complex, because of the presence of several subsets of DC and interactions with the microenvironment. In particular, DC residing in mucosal interfaces are exposed to immuno-suppressive micro-environment, and both their phenotype and their responses to immune adjuvants may be different. In this study we isolated tonsil DC, investigating their maturation and immune-activation in short-term cultures.

Tonsil APC, depleted of T and B lymphocytes, consist of monocytes, macrophages, and several subsets of myeloid and plasmacytoid DC. We compared the phenotype and responsiveness of the CD11c+/HLA DR^hi ^sub-population from tonsil, the characteristic phenotype of activated myeloid DC and blood monocyte-derived DC. We found that the differences between these two populations were considerable, and that fresh tonsil DC have a suppressed phenotype compared to freshly-generated monocyte-derived DC. Tissue-residing DC are supposed to be immature, nevertheless the expression of DC activation markers (HLA class I, HLADR, CD40, CD80, CD86), on the surface of tonsil CD11c+/HLA DR^hi ^was distinguishably weak in comparison with laboratory-generated DC. In addition, CD123+ plasmacytoid DC made up a significant fraction in the total tonsil DC population. The culture of tonsil DC in media alone induced some activation perhaps reversing immunosuppression of tonsil DC by their microenvironment, although it is also possible that the isolation procedure and culture conditions had some effect. Tonsil DC and blood monocyte-derived DC responded differently to the immune adjuvants, CD40L, IFNγ, LPS and Poly IC, each of which are potent immune stimuli for monocyte derived DC. These stimuli induce both a significant increase in the expression of surface antigens necessary for antigen presentation (surface expression of HLA class I and HLA-DR as well as co-stimulatory molecules CD80 and CD86) and production of IL12p70.

Despite the significant proportion of myeloid DC in the tonsil DC fraction, none of the adjuvants used in our study, or the most powerful combination of them, resulted in the high levels of antigen presenting and co-stimulatory molecules seen using monocyte derived DC. Since the total tonsil DC population and plasmacytoid CD123+ DC reacted similarly to the CD11c+/HLA DR^hi ^DC, we suggest that the relative unresponsiveness is not a direct result of modulation by an immunosuppressed subset of DC but an intrinsic feature of cells residing in peripheral tonsil. Little is known about interactions between different subsets of DC, although some evidence exists for the enhancement of antigen stimulation by an interplay between plasmacytoid and myeloid DC in autoimmune diseases and HIV infections [26,27], but to our knowledge the down-regulation of antigen presenting abilities of myeloid DC by a tolerogenic subset has not been demonstrated, and this area requires further investigation.

In contrast to the phenotypic data, adjuvant activation did induce some secretion of IL12p70, as measured by ELISpot. In accordance with previous studies of DC from blood and tissues by other laboratories, we found that LPS, anti-CD40 antibody and IFNγ induced expression of IL12p70, both alone and in combination. Although LPS did not greatly promote DC activation on its own, it acted synergistically with anti-CD40 antibody. Stimulation of CD40 by CD40L was reported as the most potent DC stimulus for IL12 production, with IFNγ as a strong co-stimulatory signal [2]. In this study, IFNγ was the most potent single inducer of IL12 p70, and this was unaffected by anti-CD40 antibody. This may suggest the existence of an IL12 induction pathway that is independent of CD40 signalling.

One unexpected finding was that Poly I:C inhibited the maturation and activation of tonsil DC, both in terms of IL12p70 secretion and surface antigen expression. The suppressive effect of Poly I:C was similar whether it was used as a single agent or in combination with other stimuli, even with the most potent combination of anti-CD40 antibody, IFNγ and LPS. This contradicts the belief that IFNγ stimulation cannot be reversed [28]. It should be noted that Poly I:C was reported to be a potent inducer of inflammatory cytokine expression in pure myeloid DC, generated in a laboratory, but the tonsil DC in this study were a mixed population, predominantly myeloid and plasmacytoid DC. In this case, Poly I:C not only reversed the stimulation achieved by other agents, it acted as inhibitory molecule *per se*. A similar finding was recently reported by others in murine models of autoimmune disorders [29-31]. The main Poly I:C transduction pathway, TLR3/TLR4 activation of IkkE, leads to the maturation of blood precursor derived "myeloid-like" DC. Poly I:C can act however via intracellular mechanisms omitting TLR [32]. In myeloid DC the TLR-induced production of inflammatory cytokines is entirely mediated by MyD88 [32] and it is therefore unlikely that Poly I:C influences this process. The interaction of dsRNA and plasmacytoid DC is usually not considered to occur as they lack the TLR3 on their surface [33]; but the possibility that Poly I:C acts in a TLR3-independent manner cannot be excluded.

Another possibility is that Poly I:C is too potent an activator of inflammatory responses, and its ability to mature DC is susceptible to crossover effects from other regulatory mechanisms. Genes such as IRAK-M, SOCS-1, MyD88s, SIGGIRR and ST2 modulate TLR evoked responses, and SOCS-1 directly down-modulates TLR signalling pathways, especially in response to LPS [32]. Control by these genes is exerted mainly by negative feedback, so that strong stimulation by Poly I:C would result in silencing of gene expression. Since Poly I:C shares pathways with LPS and possibly with IFNγ, it could reverse their effect by pathway "overloading". It does not affect the CD40 pathway, until NFkB is activated late in the TLR-mediated intracellular pathway [32].

Poly I:C has been used in a number of studies as a potent inducer of DC maturation to assisting the activation of T lymphocytes and has even been tested in clinical trials [13,15,16,34]. Our study shows, however, that when applied to tonsil DC, Poly I:C silences the immune response instead of eliciting it. This finding might be important for DC treatment strategies as it could be used to induce selective maturation of blood-derived DC, which might lower the risk of triggering autoimmune diseases.

In this paper we have concentrated on FACS analysis of the DC derived from tonsils, and were only able to study the secretion of one cytokine, IL-12. As DC can secrete many cytokines, including IL12, IL15, IL18, IL10, and TGFβ, which can affect the type of T-lymphocyte response induced, it would be valuable to study the effect of adjuvants on the production of other cytokines by tonsil-derived DC. New methods are now available to do this with small numbers of cells, and we hope to follow up this study accordingly. It would also be useful in future to study the effect of T-cell activation by tonsil-derived DC in co-culture, including analysis of supernatants and examination of T cell proliferation. We believe, that co-cultures of tonsil derived DC with autologous T lymphocytes will prove to be a useful model system for investigation of immune response in peripheral tissues.

## Conclusion

Tissue resident DC play a vital part in systemic immune responses, and their reaction to treatment must be taken into account when attempting immunotherapy. Dendritic cells in tissues, lymph nodes, tumours and sites of inflammation are not a homogenous population but contain myeloid and plasmacytoid DC subsets at various stages of maturation [35-39]. Dendritic cells in tissue can migrate to lymph nodes to orchestrate systemic immune responses. They have contact with local infections, allergens or tumours, and can be profoundly modulated by the local environment [40,41]. We have shown, that responses of mixed tonsil DC can be different to those of monocultures of blood derived DC, commonly used as a model in antigen presentation and cancer vaccine studies. However technically complex, extended studies on the biology of tissue resident DC, the mechanisms of interaction with T lymphocytes and molecular pathways involved in DC activation might be of considerable importance for the management of many diseases, including cancer, where they are a source of local tolerance and lead to immunosuppression of draining lymph nodes [39,42,43].

## Methods

### Tissue and ethics

Fifteen fresh tonsils were obtained after tonsillectomy performed for recurrent tonsillitis. Informed written consent was obtained from each patient (or a parent/guardian when necessary) prior to surgery, in accordance with approval by the Portsmouth and South East Hampshire Health Authority Research Ethics Committee. Human buffy coats were obtained from the National Blood Transfusion Service, Colindale, London

### Isolation of DC

Tonsils were chopped finely in a Petri dish, resuspended in wash medium (RPMI +1 % L-glutamine + 2% Pen/Strep + 1% Gentamycin; Sigma, Dorset, UK), and ground through a mesh 60 tissue grinder kit (Sigma, Dorset, UK) and then through a mesh 200 tissue grinder kit (Sigma, Dorset, UK) to obtain a single cell suspension. Isolated cells were washed twice and separated by density centrifugation on a Ficoll gradient (Histopaque-1077, Sigma, Dorset, UK), spun at 600 G for 30 min in bench top centrifuge. Cells from the Ficoll – medium interface were collected as a tonsil-derived mononuclear cell fraction, and washed twice to remove any high density medium. The mononuclear cells were resuspended at 2 × 10^8^/ml in Optiprep density gradient medium (Optiprep: Axis-Shield, Norway), made up as a working solution 1:4.2 with wash medium and overlaid with FBS. Cells were spun at 600 G for 15 min, and cells from the medium-FBS interface collected as a B-lymphocyte rich APC fraction. Cell depletion using microbeads was used to remove contaminating CD19+ B and CD3 positive T lymphocytes. (Miltenyi Biotec, Bisley, UK), accordingly to the manufacturer's protocol. Briefly, the cell pellet was resuspended in 100 μl of MACS staining buffer (PBS + 1% BSA + 0.2 mM EDTA; Sigma, Dorset, UK degassed) and 100 μl of CD3 and CD19 conjugated beads and FcR blocking reagent were added for each aliquot of 10^8 ^cells. Cells were incubated in 4°C for 15 min, and washed in the staining buffer. The cell suspension was loaded onto a MACS LD column and the negative fraction collected as tonsil DC.

### Generation of DC from blood monocytes

Peripheral blood mononuclear cells (PBMC) were isolated by density gradient centrifugation of buffy coat blood preparations over lymphoprep (Axis-Shield, Norway). The mononuclear fraction was diluted to 2 × 10^6^/ml in RPMI-1640 supplemented with 10% FCS and the monocytes allowed to adhere to plastic Petri dishes for 1 hour at 37°C. The adherent cells were removed using a cell scraper and contaminating T lymphocytes and B lymphocytes removed by MACS depletion using CD3 and CD19 microbeads respectively, as detailed above. The purified monocytes were adjusted to 1.0 × 10^6^/ml in RPMI supplemented with 5% FCS, antibiotics, 2 mM L-glutamine (all from Sigma, Dorset, UK) and containing GM-CSF (50 ng/ml) and IL-4 (10 ng/ml; both from Peprotech EC Ltd, London). The cultures were supplemented with GM-CSF and IL-4 every second day. After five days in culture, the non-adherent cells were recovered for use as immature dendritic cells.

### Adjuvant activation of DC

Cells were resuspended in culture buffer (RPMI + 10% FBS +1 % L-glutamine + 2% Pen/Strep + 1% Gentamycin; Sigma, Dorset, UK + 0.2% metronidazole) at a concentration of 2 × 10^6 ^cells/ml and 100 μl of cell suspension was added to each well. All of the adjuvants used in the study were pre-titrated in the laboratory and used at optimal concentrations similar to those used by other investigators. The final concentrations used were: Poly I:C (endotoxin free, Sigma, Dorset, UK) 25 μg/ml; LPS (Sigma, Dorset, UK) 20 μg/ml; anti-CD40 monoclonal antibody (endotoxin free, a generous gift of Professor M.J. Glennie, University of Southampton) 10 μg/ml; IFNγ (endotoxin free, Immunkin, Boehringer Ingelheim, Berkshire, UK) 1000 U/ml. The cells plus adjuvants were cultured in 5% CO_2_, at 37°C for 38–42 hrs and then collected for further analyses.

### IL12 p70 ELISpots

To assess IL12-p70 expression, tonsil DC were cultured with adjuvants in Mabtech ELISpot plates, (Mabtech, Sweden) according to the manufacturer's protocol. The plates were ethanol-pretreated and coated with monoclonal antibody at 150 μg/ml and incubated at room temperature for 6 hrs. Adjuvants at double concentrations were prepared in the 96 well Mabtech plates and 100 μl of cell suspension at 4 × 10^5 ^cells/ml was added to each well. The final adjuvant concentrations were the same as those detailed above. After 38–42 hrs of culture (5% CO_2_, 37°C) cells were washed off with PBS, detection antibody applied (1:1000 in PBS + 0.5% FBS, 100 μl per well) and the plate was incubated for 1 hr at 37°C. The detection antibody was then washed off with PBS and 100 μl of streptavidin-conjugated horse peroxidase added to each well. After 1 hr of incubation, at room temperature, the plate was washed, and 100 μl of freshly-filtered ready-made substrate added to each well. The plate was developed until the visible spots emerged or for 30 min.

### Flow cytometric analysis

Cells were collected at each step of the isolation procedure and after cultures with adjuvants. The cell concentration was adjusted to 2 × 10^6 ^cells/ml in PBSA (PBS + 0.2% BSA + 0.02% NaN3; Sigma Ltd). Fc receptor binding was blocked with 1 μg/ml of purified mouse IgG polyclonal antibody (1.0 μg/ml; Sigma, Dorset, UK. Fluorochrome-conjugated monoclonal antibodies were added into each 100 μl of cell suspension. To assess fraction purity, we stained the cells with CD19-PE, CD3-PECy5, CD14-FITC, CD123-PE, and CD11c-PECy5 (Becton Dickinson, UK). For activation status and maturity we used HLA class I – FITC, HLA DR-PE, CD40-FITC, CD80-FITC, CD83-FITC, CD86-FITC, all in combination with CD11c-PECy5 and HLA DR-PE or CD123-PE and HLA DR-PECy5 (Becton Dickinson, UK). All antibodies were used at pre-titrated, optimal concentrations. As a negative control we used a cocktail of IgG's conjugated to FITC, PE and PE/CY5 (Dako, UK). Cell suspensions were incubated at room temperature for 10 min, washed in PBSA, and fixed in 2% formalin in PBSA. Cells were analyzed in a Becton Dickinson FACSCalibur flow cytometer.

## Competing interests

The author(s) declare that they have no competing interests.

## Authors' contributions

MEP and NJB performed the experimental work, analyses and interpretation of data, drafted the manuscript and were involved in the conception and design of the study. FGG was involved with analysis and interpretation of data and made substantial contributions to design of the study. MJJ and IAC made substantial contributions to conception and design of the study, and were involved in critically revising the manuscript. All authors read and approved the final manuscript.
